# Soluble CD72, is a T-cell activator probably via binding to CD6 in homeostasis and autoimmunity

**DOI:** 10.3389/fimmu.2024.1367120

**Published:** 2024-07-04

**Authors:** Nasren Eiza, Adi Sabag, Ofra Kessler, Elias Toubi, Zahava Vadasz

**Affiliations:** ^1^ The Proteomic Unit, Bnai Zion Medical Center, Haifa, Israel; ^2^ The Rappaport Faculty of Medicine, Technion, Israel Institute of Technology, Haifa, Israel

**Keywords:** soluble CD72, CD6, T cells, autoimmunity, cytokines, signaling

## Abstract

**Background:**

CD72 is a highly required regulatory molecule in B cells. Its sufficient expression is crucial for maintaining self-tolerance. In contrast, soluble CD72 (sCD72) is reported to be increased in the serum of autoimmune diseases such as systemic lupus erythematosus and primary Sjogren’s syndrome (pSS).

**Objective:**

We wanted to assess the biological effect of sCD72 on CD4^+^T cells.

**Methods:**

We performed mass spectrometry and co-immunoprecipitation experiments to look for a sCD72 receptor on activated CD4^+^T cells. Afterward, to explore the biological functions of sCD72, we used flow cytometry for the cytokine secretion profile, a phosphorylation assay for the signaling pathway, and a CFSE dye-based assay for cell proliferation.

**Results:**

We found and validated the sCD72 and CD6 interaction as a possible ligand-receptor interaction. We also demonstrated that sCD72 significantly increases the expression of pro-inflammatory cytokines, namely IL-17A and IFN-γ, in activated CD4^+^T cells and increases the proliferation of CD4^+^T cells, possibly through its activation of the SLP-76-AKT-mTOR pathway.

**Conclusion:**

The sCD72-CD6 axis on activated CD4^+^T cells is probably a new signaling pathway in the induction of immune-mediated diseases. Therefore, targeting sCD72 may become a valuable therapeutic tool in some autoimmune disorders.

## Introduction

CD72, a fundamental regulatory receptor on B cells, down-modulates B- cell receptor (BCR)-induced signal transduction and diminishes B lymphocyte proliferation. CD72 is a member of the C-type lectin superfamily, and is expressed on the surface of all B cell stages, from the pro-B through the mature B cell stage. It carries an immune receptor tyrosine-based inhibitory motif (ITIM), which has been shown to recruit the tyrosine phosphatase SHP-1 and negatively regulate cell activity, likely by setting a threshold for BCR signaling (1–4). In a previous study, decreased CD72 expression was shown to be associated with IgG switching and SLE disease severity (5). In a subsequent study by our group, CD72 expression on the activated B cells of SLE patients was significantly lower than that of the normal controls. The lower expression of CD72 was correlated with SLE disease activity, lupus nephritis, anti-dsDNA antibodies, and low levels of complement (6). Striving to understand the mechanism by which CD72 negatively regulates auto-reactive B-cell responses, it was shown that CD72 specifically recognizes the RNA-containing endogenous TLR7 ligand Sm/RNP by its extracellular C-type lectin like-domain, thereby inhibiting B-cell responses to Sm/RNP by ITIM-mediated signal inhibition. This suggests that CD72 inhibits the development of SLE by suppressing TLR7-dependent B- cell responses to self-antigens (7, 8).

In addition to being a surface regulatory molecule on B cells, we were the first to report on the soluble form of CD72 (sCD72) in peripheral blood. In this paper, we demonstrated that serum levels of sCD72 were increased in SLE patients compared to both rheumatoid arthritis patients, as a disease control, and healthy individuals. The soluble CD72 level was significantly higher in SLE patients with renal involvement than in patients without, and the higher levels of sCD72 were correlated with SLE-related autoantibodies (9). Another study on primary Sjogren’s syndrome (pSS) reported that the soluble CD72 serum levels in patients were higher than those found in the healthy controls (10, 11).

CD4^+^T cells are pivotal to developing immune inflammation and cell activation in SLE patients as they produce higher levels of inflammatory cytokine. Though not being a canonical cytokine in SLE, however, IL-17A is considered to be important in the pathogenesis of SLE and of low regulation properties (12). Thus, one of today’s main goals is to understand the mechanisms that drive CD4^+^T cell abnormality in SLE and target it as a new therapeutic approach (13). However, the effect of soluble mediators, which can affect the CD4^+^T cells in driving autoimmunity, is still ill-defined. Therefore, we designed this study to investigate the biological role of soluble CD72 on CD4^+^T cells and its possible signaling pathway.

## Materials and methods

### Generation of soluble CD72 construct

The sCD72 construct was generated by the amplification of the extracellular part of CD72 (amino acids 117–359) from the full-length human CD72 cDNA templet (clone: 5226648, accession: BC030227, BI: 835399, GE Healthcare Dharmacon, Inc) using the following primers:

**Table:** 

sCD72–8HIS-EcoRV	5’ATGATATCCATCATCACCATCACCATCACCATCGC TATCTGCAGGTGTCTCA-3’
sCD72-XhoI	5’-ATCTCGAGCTAATCTGGAAACCTGAAAG-3’

The PCR product was cloned into a NSPI lentiviral vector in a frame with N-terminal signal peptide of human plexin-A4 and V5 tag and 8xHIS (kindly given to us by Dr. Gal Akiri, from the Mount Sinai School of Medicine, in New York City). The cloning was performed after linearizing the plasmid with EcoRV and XhoI, using T4 DNA Ligase (M0202S, New England Biolabs) according to the manufacturer’s protocol.

The soluble CD72 construct was then inserted into HEK-293T cells (ATCC) to generate a cell line that can stably secrete sCD72 to the conditioned media (HEK-293T-sCD72).

### Purification of soluble CD72 protein

HEK-293T-sCD72 (HIS tagged) cells were grown at 80% confluence in serum-free media. After twenty-four hours, the conditioned media was collected and filtered. Next, 100 mL of conditioned media were gradually loaded onto a 2mL Ni-NTA Agarose column (30210, QIAGEN) until the last drop passed, followed by two wash steps with a 50 mM phosphate buffer pH-8 containing 100mM NaCl. Finally, the elution was performed with a 50 mM phosphate buffer pH-8 containing 100 mM NaCl and 150 mM Imidazole. The elution fractions containing the purified protein were subjected to SDS-PAGE and stained with InstantBlue Coomassie protein staining to assess the protein concentration based on BSA as a concentration standard.

### Concentration of soluble CD72 protein

HEK-293T-sCD72 (HIS tagged) cells were grown at 80% confluence in serum-free media. After twenty-four hours, the conditioned media was collected and filtered. Then 25mL of conditioned media was concentrated using 3KDa Amicon Ultra-15 centrifugal filter devices for a 25-fold concentration. The concentration was determined after InstantBlue Coomassie protein staining, with BSA as a concentration standard.

### Human CD4^+^T cell purification

Peripheral blood samples were obtained from thirteen healthy controls. The study involving human participants was reviewed and approved by the Local Helsinki Committee of the Bnai-Zion Medical Center, Haifa, Israel (0180–22-BNZ). Written informed consent to participate in this study was provided by the participants. The samples were loaded on Lymphoprep™- Ficoll gradient (07801, STEMCELL Technologies) to separate mononuclear cells and centrifuged at 800xg with no break for 30 minutes. The cells were washed and later used to separate CD4^+^T cells using anti-human MACS CD4 Microbeads (130–045-101, Miltenyi Biotec), achieving 97% purity.

### Culturing CD4^+^T cells

Purified CD4^+^T cells were activated in plates pre-coated with 10µg/ml anti-CD3 monoclonal antibody (UCHT1) (16–0038-85, eBioscience™) for four hours at 37°C and later incubated with 1 µg/ml anti-CD28 monoclonal antibody (CD28.2) (16–0289-85, eBioscience™) for 48 hours at 37°C.

### Mass spectrometry assay

Activated CD4^+^T cells were rotated with 5µg/ml purified sCD72 protein (V5 tagged) for two hours at 4°C, followed by adding 2.5 mM cross-linker BS3 (l21580, Thermo Scientific) in 20 mM sodium phosphate buffer pH-8 with 150 mM NaCl for one hour at room temperature, for quenching 1M Tris-HCl pH-7.5 solution was added to a final concentration of 20mM Tris-HCl for 15 minutes. Next, cells were washed and lysed with co-IP lysis buffer [50mM Tris-HCl pH-7.5, 150mM NaCl, 1% NP-40, 0.1% DOC+ Halt™ (a protease and phosphatase inhibitor cocktail)]. One mg of total protein was incubated with 50 µl anti-V5 tag mAb Magnetic Beads (M167–11, MBL) for two hours at 4°, followed by washing them four times in wash buffer (50 mM Tris-HCl pH-7.5, 150 mM NaCl, 0.05% NP-40). Next, the proteins were eluted twice with 40µg V5 tag peptide (ab15829, Abcam) in rotation for 20 minutes. Finally, the immunoprecipitants were subjected to SDS-PAGE and stained with silver stain (24612, Pierce^®^ Thermo Scientific). For the mass-spectrometry analysis, we used a stained SDS-PAGE gel. The gel was de-stained, and the proteins were trypsinized and analyzed by LC-MSMS using the Q Exactive Plus MS. The data were analyzed using the Proteome Discoverer software. Based on the results, only dominant proteins (with at least four identified peptides) with specific interactions (at least a five-fold difference) and those that change at least 50-fold more than the control were analyzed by the STRING web-based tool.

### Co-immunoprecipitation assay

Fifty µl of anti-V5 tag mAb magnetic beads (M167–11, MBL) were loaded with 500µl concentrated sCD72 or with a control conditioned media for one hour at 4°C, followed by loading 1µg of CD4^+^T cells lysates for one more hour at 4°C. Then, the beads were washed four times in wash buffer (50 mM Tris-HCl pH-7.5, 150 mM NaCl, 0.05% NP-40). Next, the proteins were eluted twice with 40-µg V5 tag peptide (ab15829, Abcam) for 20 minutes at room temperature, followed by 20µl of SDS-PAGE sample buffer (x1) for 10 minutes at 60°C. Finally, the elution fractions were subjected to SDS-PAGE blotted onto a nitrocellulose membrane and probed with an anti-CD6 antibody (sc7320, Santa Cruz), anti-CD72 antibody (directed against 192–237 amino acids) (ab201079 Abcam), and anti-V5 antibody (PM003, MBL). The bands were visualized using an EZ-ECL Detection kit (20–500-120, Sartorius) and an ImageQuant LAS 4000 machine (GE Healthcare). Stripping antibodies for reblotting the membrane was performed using ReBlot Plus Mild Antibody Stripping Solution (2502, Millipore) for 10 minutes at room temperature.

### CellTrace™ CFSE assay for cell proliferation

Activated CD4^+^T cells from three healthy controls were stained with 5µM CFSE dye (C34554, Invitrogen™) for 20 minutes at 37°C, then they were washed with a culture medium for five minutes at 37°C, then the cells were then centrifuged. The pellet was resuspended with a complete culture medium containing 0,1 or 10 µg/ml of sCD72 for seven days at 37°C. On day seven, cells were harvested and analyzed by flow cytometry at 488 nm excitation.

### WST-1 assay for cell proliferation

Activated CD4^+^T cells from three healthy controls were seeded in triplicates with 0,1 or 10 µg/ml of sCD72 for one to seven days at 37°C (seven plates in total) in a 100 µl culturing medium. On the day of the experiment, 10 µl WST-1 reagent (5015944001, Roche) was added to each well for two hours at 37°C. Next, the optical density (O.D.) at 450 nm was measured as an indication of cells’ count.

### Phosphorylation assay

Activated CD4^+^T cells were cultured in low-serum media (1% FCS) for 16 hours. On the day of the experiment, cells were stimulated with 2µg/ml sCD72 for 10 minutes on ice, followed by five minutes at 37°C. Then, cells were washed, and the pellets were lysed with phosphorylation lysis buffer* for 10 minutes on ice and centrifuged at 14,000xg for 15 minutes at 4°C. Sixty µg of total lysates were subjected to SDS-PAGE, blotted onto a nitrocellulose membrane, and probed with an antibody directed against phosphorylated protein. The blot was then stripped using ReBlot solution (2502, Millipore) for 10 minutes at room temperature and re-probed with an antibody directed against total protein. The band intensity was visualized and quantified using an EZ-ECL Detection Kit (20–500-120, Sartorius) and the ImageQuant LAS 4000 machine and program (GE HealthCare).

*Lysis buffer for phosphorylation assays: 50 mM Tris-HCl pH-7.5, 150 mM NaCl, 4 mM EDTA, 1% NP-40 and 1:100 Halt™ [protease and phosphatase inhibitor cocktail (78420, Thermo Scientific™)].

### Antibodies:

SLP-76 Antibody (4958, Cell Signaling).

Phospho-SLP-76 (Ser376) (D9D6E) Rabbit mAb (14745, Cell Signaling).

AKT1/2/3 Antibody (H-136) (sc-8312, Santa-Cruz).

Phospho-AKT (Ser473) (D9E) XP^®^ Rabbit mAb (4060, Cell Signaling).

Phospho-mTOR (Ser2448) (D9C2) XP^®^ Rabbit mAb (5536S, Cell Signaling).

mTOR (7C10) Rabbit mAb (2983S, Cell Signaling).

### Flow cytometry staining

Activated CD4^+^T cells were incubated with sCD72 concentrated media for 48 hours at 37°C. On the day of the experiment, cells were harvested, washed, and stained with fluorescence antibodies directed against extracellular markers for 30 minutes at room temperature. Then, cells were fixed with Fix and Perm Medium A for 10 minutes, followed by a wash and permeabilization with Fix and Perm medium B (GAS004, Invitrogen) with the addition of fluorescence antibodies directed against intracellular markers for an extra 30 minutes at room temperature. The NAVIOUS EX Flow Cytometer (Beckman Coulter) analyzed the stained cells, and the results were analyzed using Kaluza, Flow Cytometry Analysis Software 2.1 (Beckman Coulter).

### Antibodies for extracellular markers

APC-Cy™7 Anti-Human CD69, 557756, BD Pharmingen™.

FITC Anti-Human CD4, A07750, Beckman Coulter.

PE-Cy7 Anti-Human CD69, A80710, Beckman Coulter.

Pacific Blue Anti-Human CD4, A82789, Beckman Coulter.

### Antibodies for intracellular markers

PE Anti-Human IFN-γ, 340452, BD FastImmune™.

PE Anti-Human IFN-γ, 506507, BioLegend.

PerCP-Cy™5.5 Anti-Human IL-17A, 560799, BD Pharmingen™.

### Statistical analysis

Means obtained from each group were compared using one-way ANOVA followed by the Kruskal-Wallis multiple comparison post-test. The following designations were used in the figures: *: p<0.05, **: p<0.01, ***: p<0.001, ****: p<0.0001 and non-specific: ns.

The statistical tests and the graphs were performed using Graph Pad Prism software.

## Results

### Soluble CD72 construct

**Figure 1:**
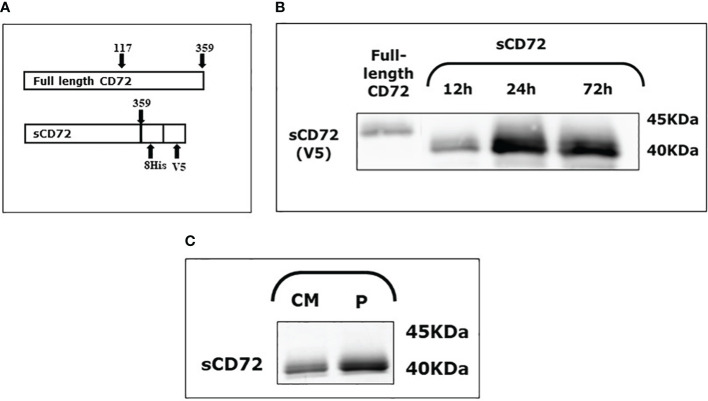
Soluble CD72 (sCD72) construct and secretion. **(A)** The sCD72 sequence contains the extracellular region of CD72 (amino acids 117–359 of the full-length CD72). **(B)** Conditioned media of HEK-293T-sCD72 cells were collected at different time points (12, 24, and 72 hours) and analyzed by western blot using an antibody directed against the V5-tag of sCD72. **(C)** Conditioned media collected from HEK-293T-sCD72 cells and purified protein were subjected to WB with specific anti-V5 antibody. sCD72, soluble CD72; h, hours; CM, Conditioned media; P, Purified protein.

CD72 is a 359- amino acid type 2 protein that contains a C-terminus extracellular domain. With the aim of generating only the extracellular part of CD72, we amplified the C-terminus region from amino acid number 117 to 359. This construct was also fused with 8xHis and V5 tags and cloned into a NSPI plasmid (**Figure 1A**, **Supplementary Figure S1**). This construct was later inserted into HEK-293T cells to generate a cell line that stably secrete a soluble form of CD72 to the media (sCD72).

To explore the sCD72 secretion kinetics, the conditioned media of HEK-293T-sCD72 was collected at different time points and analyzed by western blot. The analysis of sCD72 secretion revealed that 24 hours is the optimal time for media collection, and the secreted protein size is approximately 40KDa (**Figures 1B, C**).

### CD6 is a potential receptor of sCD72 on CD4^+^T cells

**Figure 2:**
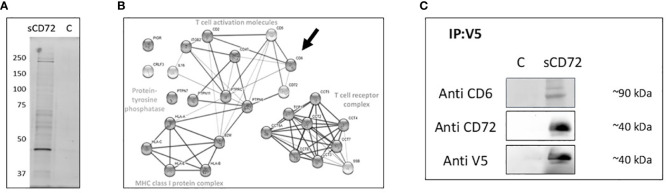
CD6 is a potential receptor for soluble CD72 on CD4^+^ T cells. **(A)** CD4^+^T cells were stimulated with 5µg/ml purified sCD72-V5 in the presence of 2.5 mM BS^3^ cross-linker, cell lysates were incubated with V5-tag mAb magnetic beads, and the eluted proteins were loaded on acrylamide gel that was later stained using silver stain. **(B)** The results of LC-mass-spectrometry presented as STRING web-based analysis of the dominant proteins eluted with sCD72 compared to the control. Proteins were divided into four main complexes: The T- cell receptor complex, the MHC class one-protein complex, T- cell activation molecules, and the protein tyrosine phosphatase. **(C)** CD4^+^T cells were stimulated with concentrated conditioned media of sCD72-V5 (sCD72), or control media (c). Cell lysates were then prepared and immunoprecipitated using V5-tag mAb magnetic beads. Blots prepared from the immunoprecipitates were subsequently probed with antibodies directed against CD6, CD72 (extracellular domain), and V5. Shown are the resulting western blots of one of three independent experiments.

Aiming to find a potential receptor of sCD72 on CD4^+^T cells, we performed a blind co-IP experiment in which we incubated CD4^+^T cells with sCD72/V5- tagged protein in the presence of the cross-linker BS^3^ and loaded the elution fractions in acrylamide gel that were later stained with silver stain. We observed two bands with predicted molecular weights of ~40kDa and ~200kDa that were specifically precipitated with sCD72 but not with the control and may point to potential sCD72 receptors (**Figure 2A**).

One of our candidates was CD100/Sema4D because of its established activation role and its well-known interaction as a ligand for CD72 receptors on B cells (14). At this juncture, antibodies targeting the V5 epitope tag of sCD72 could not immunoprecipitate CD100 from CD4^+^T cells, and we could not prove the hypothesis that CD100 could function as the receptor of sCD72 (**Supplementary Figure S1**).

Therefore, we decided to try to find the potential receptors by sending the acrylamide gel lines to LC-mass-spectrometry. The LC-mass-spectrometry analysis showed more than one dominant protein that has at least a 50-fold more significant interaction in the sCD72 sample than the control. The analysis of all proteins using the STRING web-based tool revealed that the proteins belong to four main complexes: The T- cell receptor complex (e.g. SSB), the MHC class I protein complex, T- cell activation molecules (e.gCD6, CD47, ITGB2), and the protein tyrosine phosphatase (e.g. PTPRC). Among the T- cell activation molecules, the CD6 molecule has the highest binding potential and the closest functional link to CD72 (**Figure 2B**). To validate the sCD72-CD6 interaction, we incubated activated CD4^+^T cells with sCD72 concentrated conditioned media and performed a co-IP experiment with anti-V5 beads followed by western blot analysis using an antibody directed against CD6. The results demonstrate that CD6 co-immunoprecipitated with sCD72 and may serve as a receptor for sCD72 (**Figure 2C**).

The mass spectrometry analysis indicated CD47 as a candidate receptor for sCD72, but we could not prove the specificity of the binding in a co-IP validation experiment (**Supplementary Figure S2**).

### sCD72 induces proliferation of CD4^+^T lymphocytes

**Figure 3:**
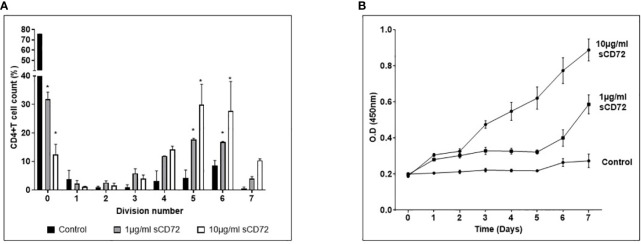
Soluble CD72 induces proliferation of CD4^+^ T cells. **(A)** Activated CD4^+^T cells stimulated with 0,1 or 10µg/ml of sCD72 were stained with CFSE dye for 7 days. The left picture is representative flow cytometry histograms. The graph represents the summary results, the X- axis represents the number of divisions (based on dye dilutions), and the Y- axis represents the percentage of cells in the indicated division cycle. **(B)** The optical density (O.D.) of WST-1 reagent added for 2 hours to activated CD4^+^T cells stimulated with 0,1 or 10 µg/ml of sCD72 at 7 different time points. The O.D. at 450nm is an indication of cell number. N=3 independent experiments; *=p<0.05.

Following our discovery and proof of the interaction between sCD72 and CD6, we were interested in the biological significance of this interaction. First, we examined the sCD72’s ability to induce the proliferation of CD4^+^T cells. As a result, we incubated activated CD4^+^T cells from different healthy donors with 0, 1, or 10 µg/ml of sCD72 in the presence of CFSE dye for seven days. On day seven, cells were harvested and analyzed by flow cytometry. The results showed a significant proliferative effect of sCD72 on T cells (based on CFSE dye dilutions). For example, most of the cells (75%) did not divide in the control treatment, while only ~30% and ~15% did not divide in the presence of 1 and 10µg/ml, respectively. Moreover, 1µg/ml of sCD72 induced more than five cycles of divisions in ~45% of the cells, whereas 10 µg/ml of sCD72 induced such division cycles in ~70% of the cells, compared to ~15% of the cells in the control treatment (**Figure 3A**).

Next, we analyzed the cell viability and metabolic activity of CD4^+^T by using a colorimetric assay based on the WST-1 reagent. Activated CD4^+^T cells from different healthy donors were seeded in triplicates with 0, 1, or 10 µg/ml of sCD72 for seven days. The cell culture’s optical density (O.D.) was measured daily. Using this method, the results also showed that sCD72 induced proliferation and enhanced metabolic activity in CD4^+^T cells as compared to the control group starting from day one. On the fourth day, we observed a significant advantage of the 10 ug/ml over 1ug/ml of sCD72, indicating a higher proliferation potential due to a higher concentration of sCD72 (**Figure 3B**). Taken together, these results demonstrated the effect of sCD72 as a proliferation inducer of CD4^+^T cells.

### CD6-sCD72 interaction activates the SLP-76, followed by AKT-mTOR signaling pathway

**Figure 4:**
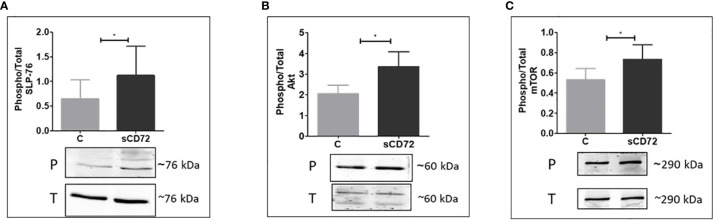
sCD72 binding to CD6 promotes the phosphorylation of SLP-76 (Ser 376), Akt (Ser 473), and mTOR (Ser2448) in CD4^+^T cells Activated CD4^+^T cells were stimulated with 2-µg/ml sCD72 for 10 min on ice followed by 5 min at 37°C. Then, cell lysates were prepared in the presence of a phosphorylation buffer, as described. Blots prepared from the lysates were probed with antibodies directed against **(A)** Phospho-SLP-76 (Ser 376), **(B)** Phospho-Akt (Ser473), and **(C)** Phospho-mTOR (Ser2448). Shown are the resulting western blots and the quantification graphs, which represent the mean ± SD of the phospho/total ratio of each indicated component from 6 independent experiments. P, phosphor; T, total; *,P<0.05.

Endeavoring to further establish the proliferation potential of sCD72, we performed phosphorylation experiments to identify which signaling pathway is activated by sCD72 in CD4^+^T cells following its interaction with CD6. In these assays, we stimulated CD4^+^T cells with sCD72 followed by western blot analysis using antibodies directed against some of the known secondary messenger molecules in T cells signaling such as ZAP-70, ERK, PLC-γ, Calmodulin, and PKC-θ (Data not shown). These experiments showed that the stimulation of CD4^+^T cells with sCD72, followed by its interaction with CD6, increased the phosphorylation of the adaptor protein SLP-76 by almost two-fold change (**Figure 4A**).

In addition, the downstream signaling molecules AKT and mTOR were also found to be phosphorylated due to sCD72 stimulation, compared to the control treatment (**Figures 4B, C**). Taken together, these results suggest that CD6 functions as a signaling transducing receptor for sCD72 in CD4^+^T cells.

### sCD72 shifts CD4^+^T lymphocytes to an inflammatory profile

**Figure 5:**
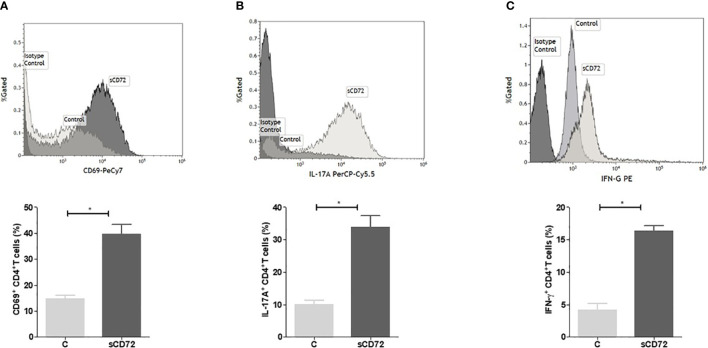
Soluble CD72 activates CD4^+^ T cells. Purified human CD4^+^T cells were stimulated with 10 µg/ml anti-human CD3 and 1µg/ml anti-human CD28 and simultaneously incubated with 1 µg/ml of purified sCD72 for 48 hours at 37°C. The flow cytometry results of **(A)** CD69 expression, **(B)** IL-17A, and **(C)** IFN-γ levels following sCD72 stimulation compared to control. N=13 independent experiments; *=p<0.05.

We incubated activated cells with 1µg/ml of purified sCD72 for 48 hours at 37°C, after which the cells were harvested and stained with antibodies directed against CD25, CTLA-4, CD40L, CD69, IL-17A, IFN-γ, and IL-10, and analyzed using flow cytometry intending to define the cytokine profile of CD4+ T cells stimulated with sCD72. The analysis showed that sCD72 significantly increased the expression of CD4^+^CD69^+^T cells (from 14.72 ± 5.28% to 39.66 ± 14.56%, *p<0.05*) (**Figure 5A**; **Supplementary Figure S3A**). The secretion levels of the pro-inflammatory cytokines IL-17A and IFN- γ also increased almost threefold due to sCD72 stimulation (**Figures 5B, C**; **Supplementary Figures S3B, C**). Interestingly, when we used a blocking antibody against CD100, we could not detect any effects on the secretion level of the above-mentioned cytokines. We validated these results by measuring the cytokine secretion in the conditioned media using a BD^®^ Cytometric Bead Array (**Supplementary Figure S3D**). In contrast, no significant changes were observed in the expression of CD40L, CD25, CTLA-4, and the anti-inflammatory cytokine IL-10 (**Supplementary Table S1**, **Supplementary Figure S4**). Therefore, we postulated that sCD72 activates CD4^+^T cells and shifts them to secrete pro-inflammatory cytokines.

## Discussion

CD72 receptor on B cells is widely reported to be one of many regulatory receptors playing role in maintaining self-tolerance and preventing autoimmune responses. The main mechanism by which CD72 maintains its regulatory property is the recruitment of SHP-1and the down regulation of BCR signaling (1–4). Soluble CD72 (sCD72) is found in peripheral blood following its shedding from activated B cells or due to its proteolytic cleavage.

Soluble CD72 was found to be higher in the serum of patients with autoimmune diseases such as SLE and Sjogren’s syndrome as compared to healthy controls. High levels of sCD72 were also positively correlated with disease severity, mainly with lupus nephritis, indicating its probable essential role in autoimmunity pathogenesis (9, 11).

In our current study, we found that sCD72 is a CD4+ T cells stimulator, which significantly increases the secretion of the pro-inflammatory cytokines IL-17A and IFN-γ, two of the classical and essential activation markers in immune-mediated diseases. Recent studies demonstrated the high relevance of IL-17A in the pathogenesis of SLE activity (15, 16). In addition, co-culturing sCD72 with CD4^+^T cells significantly increased their proliferation; strengthening the idea, that sCD72 might be a frontline player in shifting CD4^+^T cells into a pro-inflammatory status and losing their self-tolerance. The issue of sCD72 being a stimulatory rather than a regulatory molecule is similar to other bound regulatory molecules and their soluble forms. In this respect, many cleaved checkpoint molecules such as sLAG3 and sPD-L1 turned to be stimulatory and increased in many immune-mediated disorders (17, 18).

While searching blindly for a potential sCD72 receptor on T cells, with the help of high-resolution mass spectrometry, we identified multiple dominant proteins, mainly CD6, with a high specific interaction compared with the control. CD6 is a signal-transducing class one-scavenger receptor mainly expressed on T cells and works as both an adhesion and an intracellular signal-transducing receptor. CD6 co-stimulation enhances proliferative efficacy and increases a distinct gene transcription profile associated with cell activation, differentiation, and survival. Additionally, it promotes a pro-inflammatory response in T cells (19–21).

CD6 is a unique multi-ligand receptor reported to be involved in several autoimmune diseases. For example, CD6 and its ligand ALCAM (CD166) are considered to be risk factors for the neuro-autoimmune disease multiple sclerosis (MS). It is possible that these two molecules might contribute to pathogenesis by enhancing active T-cell migration across the brain barrier (22, 23). Knockout of CD6 in MS mice model (EAE) could decrease the infiltration of inflammatory T cells into the nervous system and thereby protect from developing the disease (24). Unfortunately, clinical trials using anti-CD6 monoclonal antibodies to treat MS were unsuccessful (25, 26). The same axis of CD6-ALCAM was reported to be involved in SLE disease, especially in patients with lupus nephritis. Inhibition of CD6 in SLE mice models improved renal damage and increased mice survival due to the inhibition of the inflammation process. However, this treatment failed to prevent the initiation of the disease (27). Lower CD6+ memory B cells in patients with primary Sjogren’s syndrome (pSS), was reported to be due to the transmigration of CD27+ memory B cells into salivary glands of pSS patients. This was found to be in association with the highly expressed CD166 on epithelial cells of salivary glands, suggesting that anti-CD6 antibodies could become a relevant therapeutic option for pSS (28). The knockout of CD6 in the mouse model of collagen-induced arthritis (CIA) was noticed to result in reduced levels of Th17, and serum levels of collagen-reactive total IgG. In this respect, the induction of anti-CD6 antibody was highly effective in reducing joint inflammation in CIA (29).

The effect of blocking CD6 demonstrates CD6’s value in preventing systemic inflammation reactions. However, since CD6 is highly expressed on T cells, non-specific blocking will cause a depletion in all the T cells’ subsets, which may result in severe side effects (30, 31). Therefore, a better strategy for blocking the CD6 interaction with a specific ligand would be a better approach. Therefore, one of our plans is to explore the exact domain in CD6 that binds sCD72, with the aim of synthesizing small agents that can interfere with this binding as a new strategy to block CD6.

To conclude, our observations suggest that sCD72 may be released from autoreactive B cells, resulting in high CD4^+^T cell activation, which contributes to enhanced inflammation and autoimmune reactions. We believe that CD6 might be a sCD72 receptor on T cells. A more comprehensive understanding of the sCD72-CD6 axis will allow for the development of future strategies for treating autoimmunity.

The study involving human participants was reviewed and approved by the Local Helsinki Committee of the Bnai-Zion Medical Center, Haifa, Israel (0180–22-BNZ). Written informed consent to participate in this study was provided by the participants.

## Data availability statement

The mass spectrometry proteomics data have been deposited to the ProteomeXchange Consortium via the PRIDE partner repository with the dataset identifier PXD053518 and the data is publicly available on the webpage: http://www.ebi.ac.uk/pride/archive/projects/PXD053518.

## Ethics statement

The study involving human participants was reviewed and approved by the Local Helsinki Committee of the BnaiIn review Zion Medical Center, Haifa, Israel (0180-22-BNZ). Written informed consent to participate in this study was provided by the participants.

## Author contributions

NE: Conceptualization, Formal analysis, Investigation, Methodology, Validation, Visualization, Writing – original draft, Writing – review & editing. AS: Writing – original draft, Writing – review & editing. OK: Writing – original draft, Writing – review & editing. ET: Writing – original draft, Writing – review & editing. ZV: Conceptualization, Data curation, Formal analysis, Investigation, Methodology, Supervision, Visualization, Writing – original draft, Writing – review & editing.
