# Supplementary material for: Soluble CD72, is a T-cell activator probably via binding to CD6 in homeostasis and autoimmunity
Source: Front Immunol. 2024 Jul 4;15:1367120. doi: 10.3389/fimmu.2024.1367120 (PMC11254670; doi:10.3389/fimmu.2024.1367120)
Supplement: Supplementary file 1 [file Presentation_1.pptx]

## Slide 1
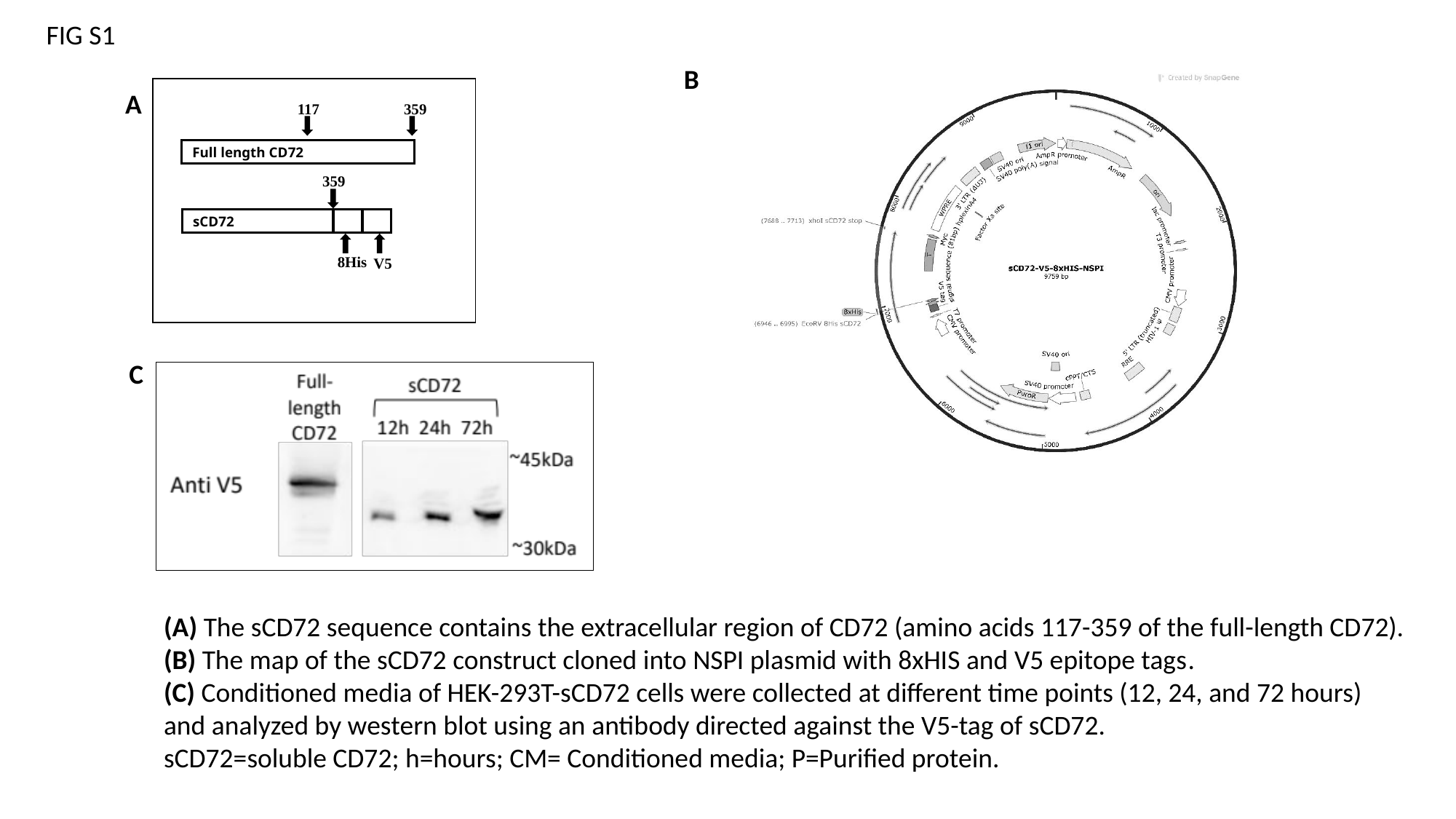

FIG S1
B
117
359
Full length CD72
359
sCD72
8His
V5
A
C
(A) The sCD72 sequence contains the extracellular region of CD72 (amino acids 117-359 of the full-length CD72).
(B) The map of the sCD72 construct cloned into NSPI plasmid with 8xHIS and V5 epitope tags.
(C) Conditioned media of HEK-293T-sCD72 cells were collected at different time points (12, 24, and 72 hours) and analyzed by western blot using an antibody directed against the V5-tag of sCD72.
sCD72=soluble CD72; h=hours; CM= Conditioned media; P=Purified protein.

## Slide 2
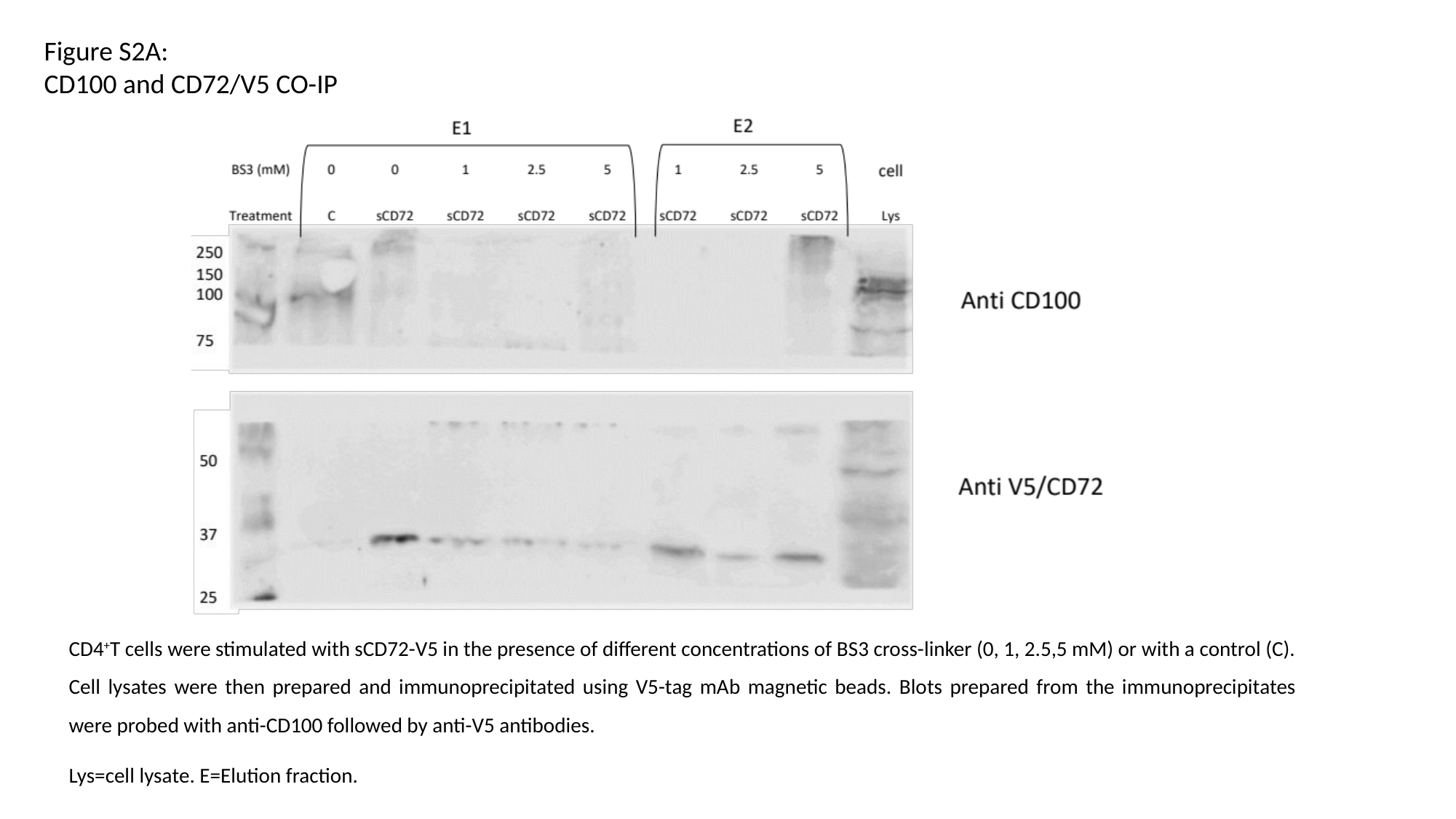

Figure S2A:
CD100 and CD72/V5 CO-IP
CD4+T cells were stimulated with sCD72-V5 in the presence of different concentrations of BS3 cross-linker (0, 1, 2.5,5 mM) or with a control (C). Cell lysates were then prepared and immunoprecipitated using V5-tag mAb magnetic beads. Blots prepared from the immunoprecipitates were probed with anti-CD100 followed by anti-V5 antibodies.
Lys=cell lysate. E=Elution fraction.

## Slide 3
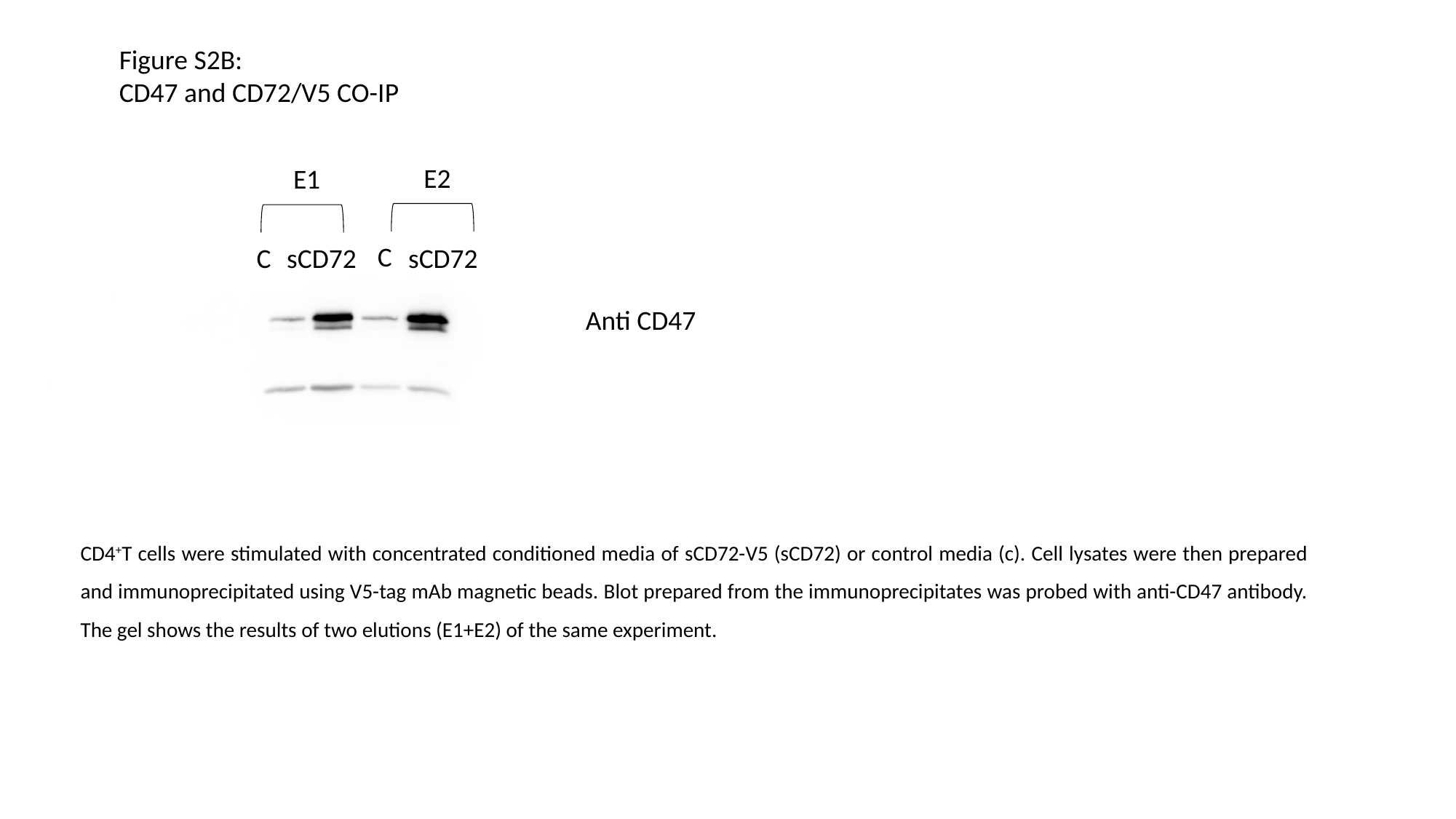

Figure S2B:
CD47 and CD72/V5 CO-IP
 E2
 E1
 C
sCD72
sCD72
 C
Anti CD47
CD4+T cells were stimulated with concentrated conditioned media of sCD72-V5 (sCD72) or control media (c). Cell lysates were then prepared and immunoprecipitated using V5-tag mAb magnetic beads. Blot prepared from the immunoprecipitates was probed with anti-CD47 antibody. The gel shows the results of two elutions (E1+E2) of the same experiment.

## Slide 4
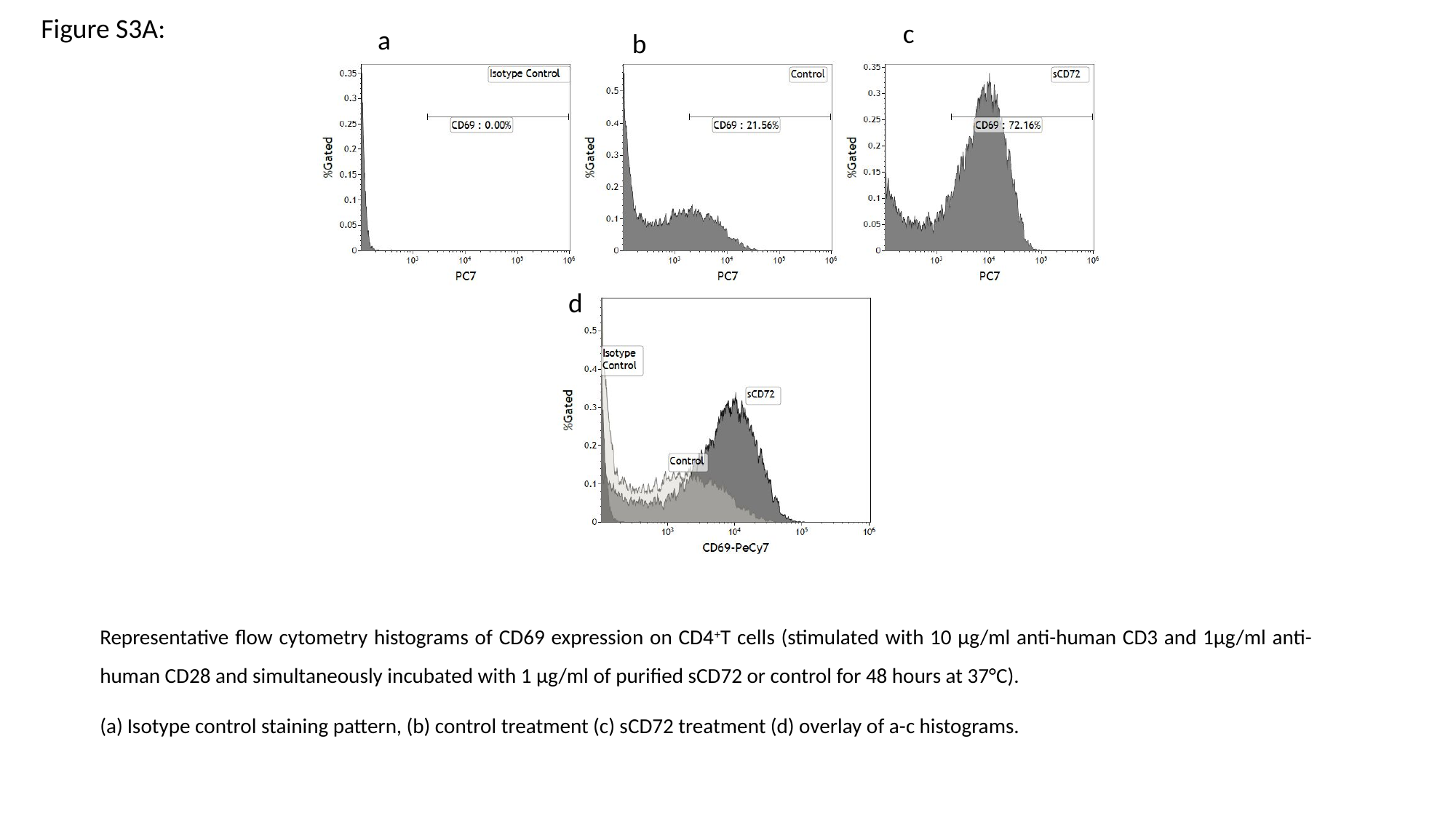

Figure S3A:
c
a
b
d
Representative flow cytometry histograms of CD69 expression on CD4+T cells (stimulated with 10 µg/ml anti-human CD3 and 1µg/ml anti-human CD28 and simultaneously incubated with 1 µg/ml of purified sCD72 or control for 48 hours at 37°C).
Isotype control staining pattern, (b) control treatment (c) sCD72 treatment (d) overlay of a-c histograms.

## Slide 5
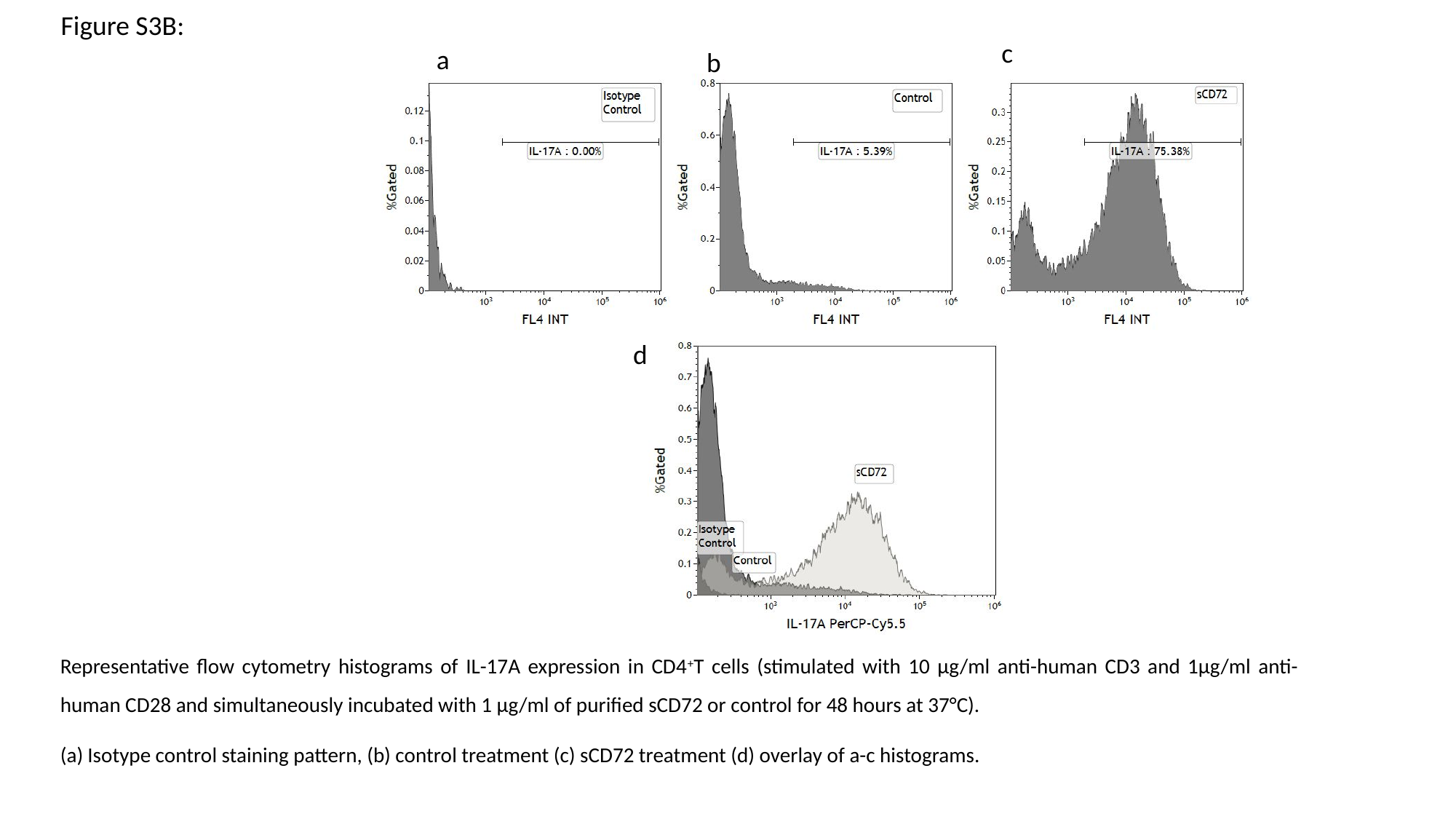

Figure S3B:
c
a
b
d
Representative flow cytometry histograms of IL-17A expression in CD4+T cells (stimulated with 10 µg/ml anti-human CD3 and 1µg/ml anti-human CD28 and simultaneously incubated with 1 µg/ml of purified sCD72 or control for 48 hours at 37°C).
Isotype control staining pattern, (b) control treatment (c) sCD72 treatment (d) overlay of a-c histograms.

## Slide 6
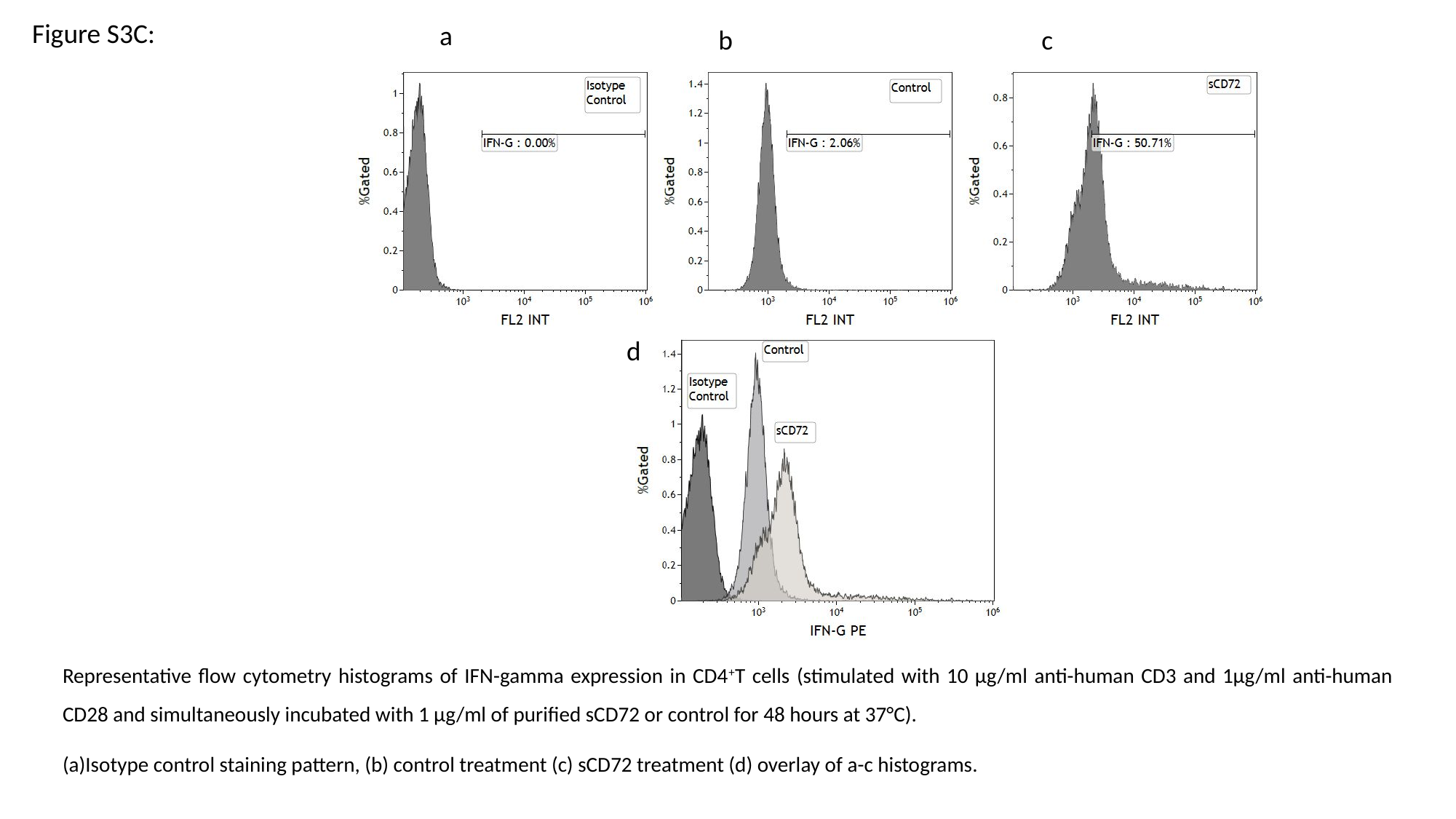

Figure S3C:
a
b
c
d
Representative flow cytometry histograms of IFN-gamma expression in CD4+T cells (stimulated with 10 µg/ml anti-human CD3 and 1µg/ml anti-human CD28 and simultaneously incubated with 1 µg/ml of purified sCD72 or control for 48 hours at 37°C).
(a)Isotype control staining pattern, (b) control treatment (c) sCD72 treatment (d) overlay of a-c histograms.

## Slide 7
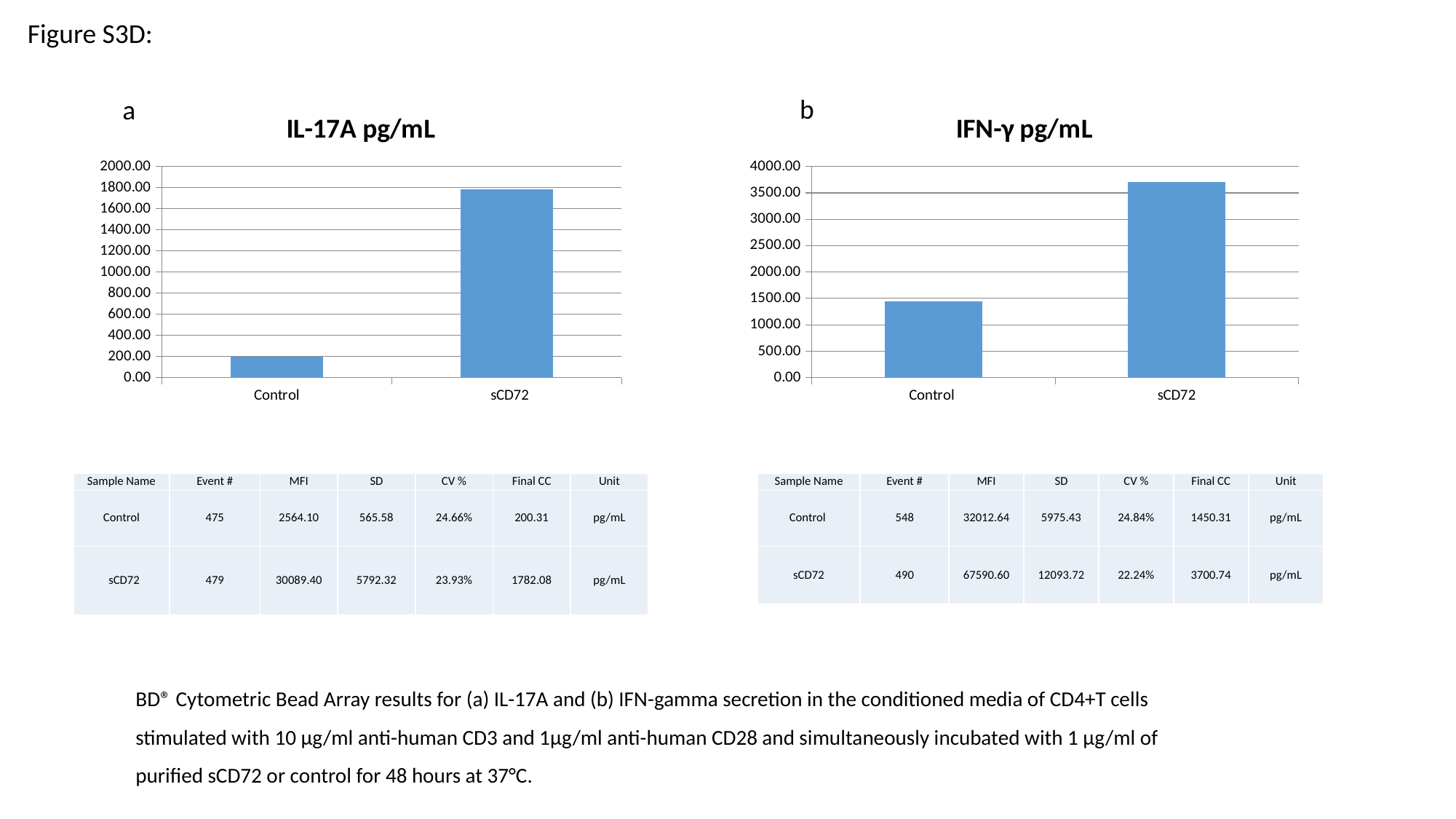

Figure S3D:
b
a
### Chart: IL-17A pg/mL
| Category | |
|---|---|
| Control | 200.3069254983039 |
| sCD72 | 1782.080917901846 |
### Chart: IFN-γ pg/mL
| Category | |
|---|---|
| Control | 1450.311867734287 |
| sCD72 | 3700.7369153415516 || Sample Name | Event # | MFI | SD | CV % | Final CC | Unit |
| --- | --- | --- | --- | --- | --- | --- |
| Control | 475 | 2564.10 | 565.58 | 24.66% | 200.31 | pg/mL |
| sCD72 | 479 | 30089.40 | 5792.32 | 23.93% | 1782.08 | pg/mL |
| Sample Name | Event # | MFI | SD | CV % | Final CC | Unit |
| --- | --- | --- | --- | --- | --- | --- |
| Control | 548 | 32012.64 | 5975.43 | 24.84% | 1450.31 | pg/mL |
| sCD72 | 490 | 67590.60 | 12093.72 | 22.24% | 3700.74 | pg/mL |
BD® Cytometric Bead Array results for (a) IL-17A and (b) IFN-gamma secretion in the conditioned media of CD4+T cells stimulated with 10 µg/ml anti-human CD3 and 1µg/ml anti-human CD28 and simultaneously incubated with 1 µg/ml of purified sCD72 or control for 48 hours at 37°C.

## Slide 8
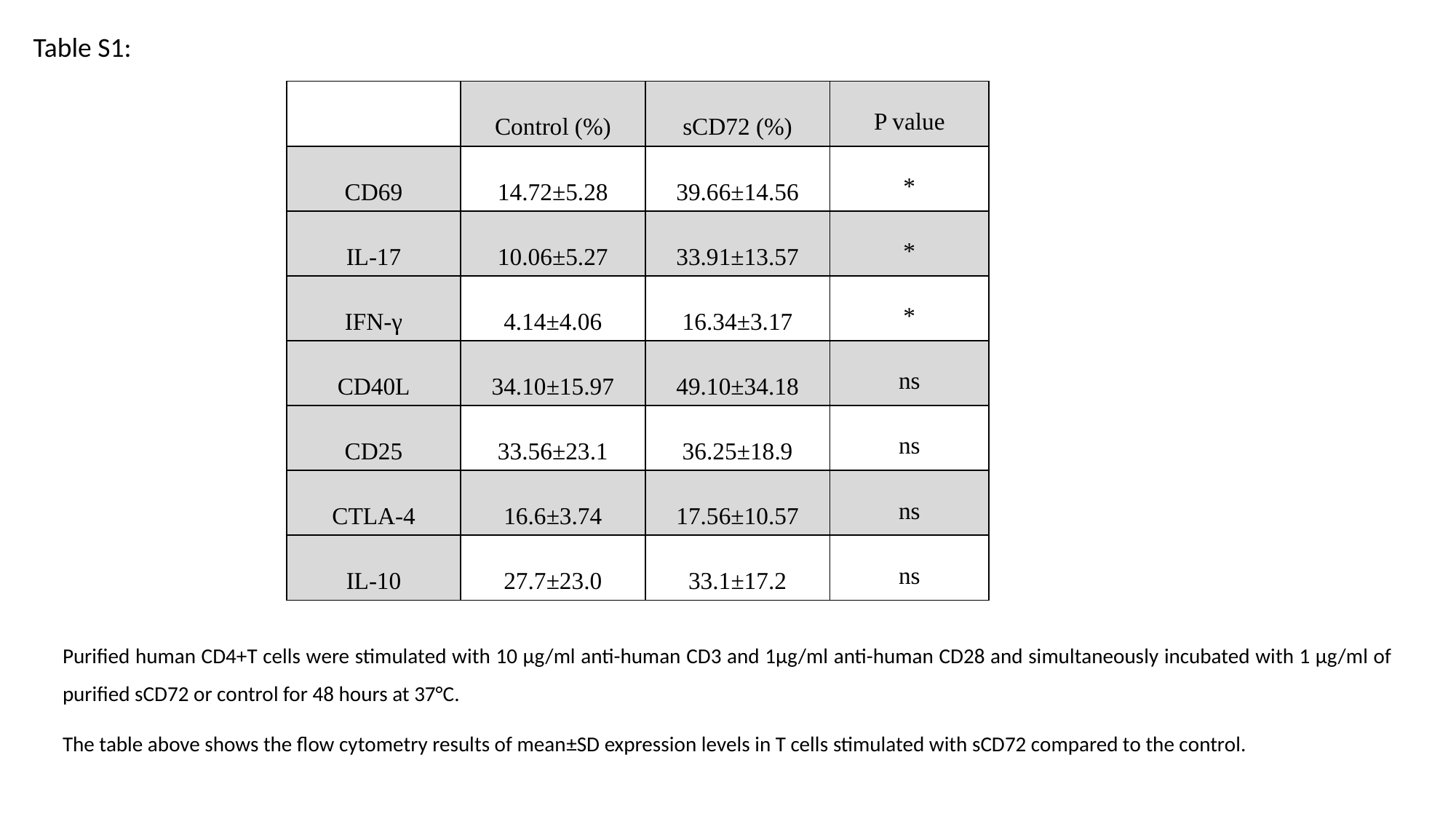

Table S1:
| | Control (%) | sCD72 (%) | P value |
| --- | --- | --- | --- |
| CD69 | 14.72±5.28 | 39.66±14.56 | \* |
| IL-17 | 10.06±5.27 | 33.91±13.57 | \* |
| IFN-γ | 4.14±4.06 | 16.34±3.17 | \* |
| CD40L | 34.10±15.97 | 49.10±34.18 | ns |
| CD25 | 33.56±23.1 | 36.25±18.9 | ns |
| CTLA-4 | 16.6±3.74 | 17.56±10.57 | ns |
| IL-10 | 27.7±23.0 | 33.1±17.2 | ns |
Purified human CD4+T cells were stimulated with 10 µg/ml anti-human CD3 and 1µg/ml anti-human CD28 and simultaneously incubated with 1 µg/ml of purified sCD72 or control for 48 hours at 37°C.
The table above shows the flow cytometry results of mean±SD expression levels in T cells stimulated with sCD72 compared to the control.

## Slide 9
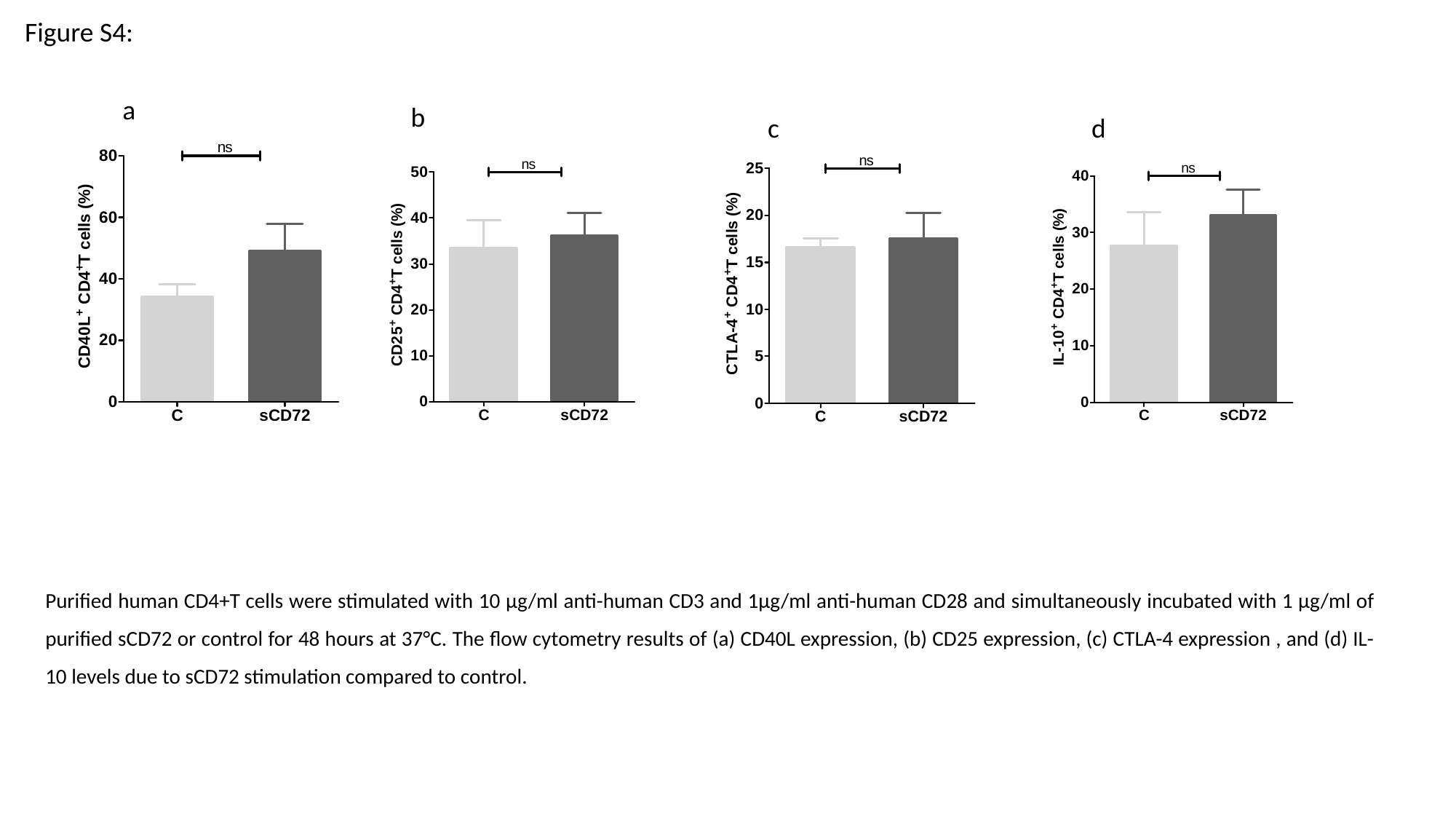

Figure S4:
a
b
c
d
Purified human CD4+T cells were stimulated with 10 µg/ml anti-human CD3 and 1µg/ml anti-human CD28 and simultaneously incubated with 1 µg/ml of purified sCD72 or control for 48 hours at 37°C. The flow cytometry results of (a) CD40L expression, (b) CD25 expression, (c) CTLA-4 expression , and (d) IL-10 levels due to sCD72 stimulation compared to control.
